# Navigating Immersion: Usability Study of a Virtual Reality Application Designed for Older Adults with Dementia and Their Caregivers

**DOI:** 10.1089/jmedxr.2024.0034

**Published:** 2025-04-11

**Authors:** Hira Alizai, Madeline Wong, Danielle Tchao, Lora Appel

**Affiliations:** ^1^ OpenLab, KITE, University Health Network, Toronto, Ontario, Canada.; ^2^ Dalla Lana School of Public Health, University of Toronto, Toronto, Ontario, Canada.; ^3^ Faculty of Arts & Science, University of Toronto, Toronto, Ontario, Canada.; ^4^ Faculty of Health, York University, Toronto, Ontario, Canada.; ^5^ Michael Garron Hospital, Toronto, Ontario, Canada.

**Keywords:** virtual reality, older adults, recreation, therapeutic, social interaction, caregivers, immersive, user experience, usability

## Abstract

A growing number of therapeutic virtual reality (VR) applications are targeted at older adults, but few are evidence-based and designed together with end-users, thus limiting the adoption of this potentially game-changing technology. There is also little research exploring VR applications developed for dyadic use between older adults and their caregivers, who are commonly a part of the safe and optimal experience. The objective of this study was to evaluate the initial usability of an application designed to make accessing immersive VR easier for older adults with dementia and their caregiver(s). This was a non-randomized, prospective mixed-methods think-aloud study that consisted of a two hour usability session with seven participant dyads (one person living with dementia and their caregiver). Descriptive statistical analyses were conducted on demographic quantitative data, and three researchers conducted thematic analysis on observation notes as well as the participants’ qualitative responses. Themes identified included headset comfort and safety, usability and navigation, quality (spanning over sub-themes of video, audio, and connectivity), and content. Our main findings highlighted success in delivering the platform’s main function as its immersive experience and ability to accommodate socialization and bonding between older adults and caregivers received positive reception from participants. Additionally, there was a need to improve the application’s audio quality, as well as streamline its navigation and enlarge interface elements for easier use. These findings provide a guide for development of future VR interventions targeted toward older adults and their caregivers, of which there is currently a lack of in the commercial market and in evidence-based research, despite their demonstrated potential.

## Introduction

Virtual reality (VR) is increasingly applied in wellness, particularly targeting older adults facing various age-related challenges. These immersive technologies aim to overcome barriers such as cognitive and mobility limitations associated with aging. For instance, VR serves as an alternative to real-world travel, enabling older adults to experience destinations they might find physically or cognitively challenging to visit due to long distances or difficulties navigating within their surroundings.^1–3^ Challenges during physical travel, such as navigating uneven terrain or encountering inaccessible facilities like restrooms and public transit, further highlight the appeal of VR as a viable option.^4–7^ Moreover, VR accommodates physiological limitations, such as limited vision or mobility issues, which can be exacerbated during traditional travel.^3,8^ Age-related cognitive impairments, including slower information processing and reduced executive function, also contribute to the interest in VR-enhanced experiences, particularly as they offer a safer and more accessible alternative.^2,3,9^ Some older adults (e.g., people with dementia) may also require the accompaniment of a support person such as an informal caregiver while traveling,^10^ which adds to its financial and logistical constraints. Consequently, VR-accommodated travel is gaining traction among researchers and health care providers, with applications such as using immersive environments to stimulate older adults complementing traditional “armchair travel” activities.^11^

Despite the increasing interest and growing number of VR applications in the market, few products are evidence-based or have been designed and tested with real users.^1,12–14^ Most academic and market research does not include individuals with vision, auditory, cognitive and physical impairment, or reflect the real challenges experienced by this population, ultimately limiting the adoption of this potentially game-changing technology.^15^ For these technologies to be successful, they must effectively meet the needs and abilities of older adults and their caregivers. Currently, many studies with this population highlight the importance of simplifying navigation, selection, and interaction within VR experiences.^1,16^ Given the market penetration of smart devices such as mobile phones and tablets—particularly following the COVID-19 pandemic—there has been a growth in comfort level, even among older adults, with using technology,^17^ which may serve as the key to improving navigation. Our research team, guided by an advisory board composed of individuals with lived experience, advocates, clinicians, and caregivers, has developed a platform designed to facilitate engagement with VR content in a head-mounted display (HMD) through a paired tablet device. This platform aims to address the specific needs and preferences of older adults, ensuring that VR experiences are accessible, intuitive, and enjoyable for this user group.

As with our previous trials relating to VR, this study followed the practices of a VR2 trial^18^ by both developing content and conducting early feasibility, acceptability, and usability testing of the VR intervention through engagement with intended users (i.e., older adults and their caregivers) and other experts.^19–25^

### Objective

To evaluate the initial usability of an application designed to make accessing immersive VR easier for older adults with dementia and their caregivers. Secondary aims include identifying areas for improvement and new features to be implemented in future iterations to continue to ensure that subsequent versions meet the needs and goals of this population. The authors aim to report on: (1) the study’s participant sample; (2) hardware: usability, headset comfort, and safety of VR; and (3) software: navigation, quality, and content.

## Materials and Methods

### Study design

This was a non-randomized, prospective mixed-methods think-aloud usability study conducted between January and February 2024. The study design consisted of a two hour VR app usability session for each participant dyad. Ethics approval for this study was obtained from York University’s Research Ethics Board (certificate #: e2023-248).

### Study procedure

Participants completed a data collection form that captured elements including demographics, participant health history using the Clinical Frailty Scale,^26^ and ratings of experience and comfort using technologies (Supplementary Appendix A1). Participants then took part in a usability test that lasted on average for 1 h. Most dyads were composed of an older adult and their caregiver, who took on the primary role of viewer and navigator, respectively. Dyads without an older adult did not have any preferred role designation.

In the role of a viewer, participants wore a Meta Quest II HMD to experience 360° virtual environments and 2D content in a 360° virtual scene, and to communicate with the navigator through the app’s two-way communication feature. Their tasks included providing input for the content they were interested in viewing, looking around in all directions (including above, below, and behind) while viewing 360° content, and voicing their observations, feelings, and other feedback aloud.

**FIG. 1.**
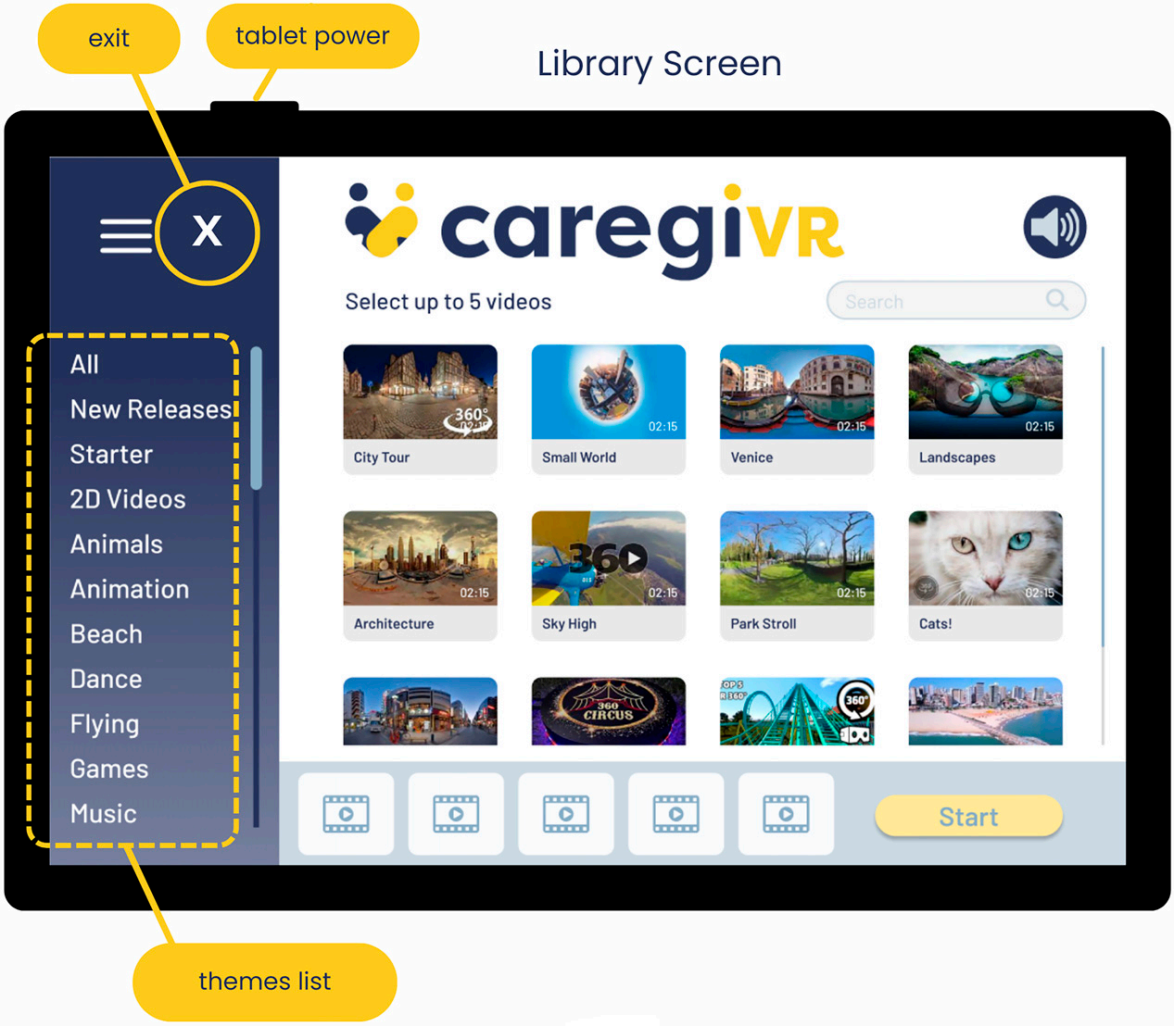
Navigator-participant (caregiver) view on paired tablet of the library page, with experiences tagged by theme.

**FIG. 2.**
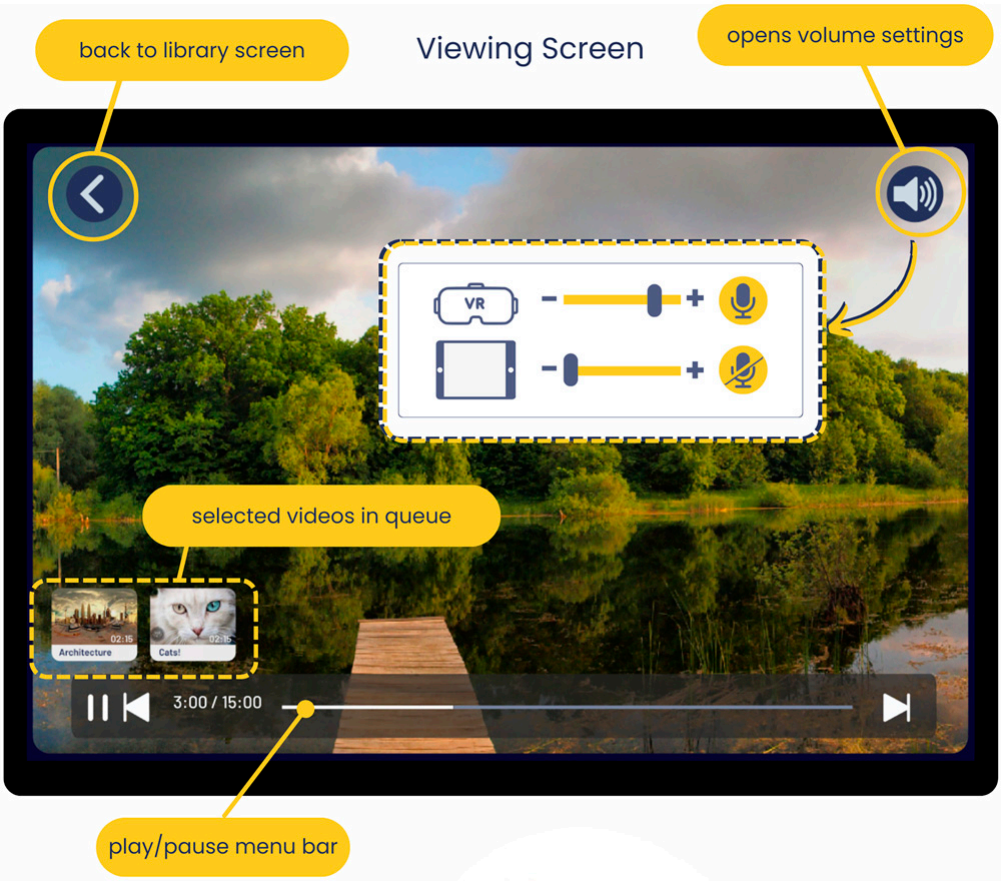
Navigator-participant (caregiver) view on the paired tablet of the experience including audio controls.

**FIG. 3.**
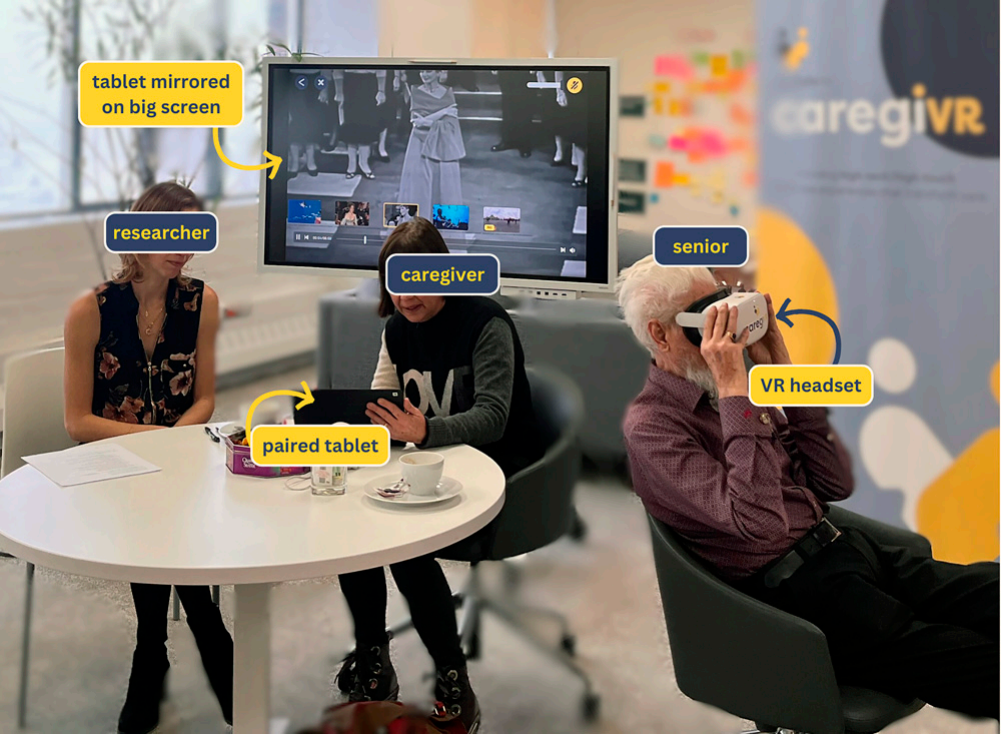
Viewer-participant (senior) experiencing VR in a headset, while the content is selected by a navigator-participant (caregiver) cast to a large screen.

In the role of a navigator, participants interacted directly with the VR app using a Samsung Galaxy S9 FE tablet. Navigators would select VR content for their paired viewer (see Fig. 1), interact with the viewer, and interact with the app’s settings (e.g., volume, pausing content, and exiting the session, see Fig. 2). Like the viewer, the navigator would also voice their observations, thoughts, and other feedback aloud throughout the usability test (see Supplementary Appendix A2). At one point during this phase, navigators were instructed to take the tablet to another room and test the two-way communication feature. Participants could also switch roles after completing all tasks in their primary roles. Figure 3 captures a viewer-participant wearing a VR headset, while a navigator-participant controls the experience from a paired tablet.

One researcher was assigned to interact with the participants by providing instructions and assisting participants if required or requested. Each participant was observed by at least one researcher who took observation notes of content selected by participants, points of challenge or intuition, points where assistance or explanation was required, participant verbal feedback, and technical issues from either hardware or software. Researchers also took note of participant body language while using the platform that demonstrated engagement, enjoyment, or reminiscence, along with apprehension/anxiety, aggression, or sadness. During some usability sessions, the tablet view was also projected on a large screen behind participants in order to provide researchers with a clear view of the content that participants engaged with. This arrangement allowed observers to take notes while at an appropriate distance from participants and did not impact the experience for participants at any point during the sessions.

After completing the usability testing phase, participants who took on a primary role as navigator completed a System Usability Scale (SUS) questionnaire (see Supplementary Appendix A1), which captured their perceived usability of the VR app. Finally, all participants took part in a semi-structured interview that captured in-depth feedback that probed audio and visual quality, elements of the app they enjoyed, elements that were challenging, inclination to recommend to others, and suggestions for improvement (see Supplementary Appendices A1 and A2).

### Data analysis

The form and questionnaire responses, along with observation notes, were manually transcribed, collated, and consolidated into a centralized digital document. Two researchers (H.A. and M.W.) conducted descriptive statistical analyses on both demographic and quantitative data. Three researchers (M.W., H.A., and D.T.) performed thematic analysis on the participants’ qualitative responses and observation notes, with peer-validation checks at each stage. Data were initially grouped into four categories: glitches, likes, complaints, and recommendations. Final categories were then coded as headset comfort and safety, usability and navigation, quality, and content by H.A. and M.W. based on an initial set of themes created by D.T.

Sub-themes of connectivity, video, and audio were created under the category of quality to facilitate future application revisions. The previously categorized data were then reassigned to these four main themes. See Table 1 for the charting framework and Supplementary Appendix A2 for the complete data.

**Table 1.** Quantitative Category and Theme Definitions

Item	Description
Categories	Nature of feedback or observation
Glitches	Technical functionality that did not operate the way it was intended or designed to, or functionality that only partially worked
Likes	Aspects of the experience that users enjoyed or found helpful
Complaints	Aspects of the experience that users struggled with
Recommendations	Feedback on altering existing features or proposing new features to improve the overall experience
Themes	Element of participant application experience
Navigation	How users move through different content, features, and functionalities within the app. This includes logging in and out, selecting and exiting content, interacting with buttons, and controlling audio and video, among other elements
Quality	*Connectivity*: The connection quality between the two devices required to use the app—the tablet and HMD—which engage in joint, synchronous communication during a session *Video*: Viewing videos, such as the display of video on a tablet and in headset or resolution quality *Audio*: Quality of the video’s audio track and quality of audio communication between navigators and viewers (volume, echoing, or competing audio between user and video)
Content	Content of videos or the range of app content available for users, such as the customization of content or incorporating educational and interactive elements

HMD, head mounted display.

Thematic analysis, commonly used in health technology assessments, was employed to systematically analyze and organize qualitative data,^27^ such as responses gathered through interview or focus group components, into themes which produce meaning from this subjective feedback.^28,29^ Similar studies have utilized thematic analysis to identify patterns in qualitative findings and use this information to address study objectives.^28–31^ The thematic analysis in this study aimed to produce distinct themes that captured the full breadth of participants’ user experiences with the immersive application. This approach was particularly suitable for capturing differences in participant perspectives and abilities, enabling the organization of findings that could inform improvements to VR accessibility for older adults and enhance application features.

## Results

### Participant sample

Supplementary Appendix A3 presents the demographics of the seven dyads (14 participants) recruited. Of the seven, one dyad (14.3%) was composed of colleagues, one dyad (14.3%) consisted of an engaged couple, one dyad (14.3%) with spouses, and four dyads (57.1%) with a parent and child. The mean age of viewer-participants and navigator-participants was 71.6 and 53.9 years, respectively. Table 2 further describes participant characteristics including mobility and cognitive barriers.

**Table 2.** Self-Reported Participant Characteristics

Participant characteristic	Number of participants (%)
Gender	
Female	7 (50.0)
Male	7 (50.0)
Primary language	
English	14 (100.0)
Dominant hand	
Right	13 (92.9)
Left	0 (0.0)
Ambidextrous (i.e., both)	1 (7.1)
Mobility barriers	
Difficulty using hands or arms	2 (14.3)
Require mobility aid(s) to get around (e.g., walker, cane, wheelchair)	4 (28.6)
Wear glasses or contacts for vision correction (e.g., reading, near, and/or distance)	10 (71.4)^a^
Wear hearing aids	2 (14.3)^b^
Cognitive barriers	
Difficulty with cognition or memory	1 (7.1)
Comfort with technology	
None to very limited	2 (14.3)
Basic or functional	4 (28.6)
Intermediate	7 (50.0)
Advanced	1 (7.1)

^a^ Five of these 10 participants (50.0%) wore glasses during study session.

^b^ Worn during study session.

### Headset comfort and safety

Overall, most participants found the headset physically comfortable, with many adjusting their posture to alleviate minor discomfort, while others felt the experience was well worth the occasional inconvenience (P13-Viewer). Three participants (21.4%) experienced mild cybersickness (e.g., dizziness, nausea) under specific conditions, such as quick head movements or fast-moving content. Four participants (28.6%) preferred using the headset with head straps, and one participant (P14-Navigator) noted potential fatigue from holding the headset. The support of a caregiver helped one frail participant (P9-Viewer) manage the headset’s weight more effectively, with most participants appearing physically comfortable throughout the experience.

### Usability and navigation

The app’s usability received positive feedback, with a mean SUS score of 72.5, indicating “good” usability.^32^ Questionnaire responses and individual SUS scores are provided in Supplementary Appendix A4. Supplementary Appendix A5 summarizes the number of responses and average ratings on a scale of 1–5 received in response to questions relating to the participants’ experience using the platform. By the second phase of the sessions, all participants were able to successfully navigate and select videos, demonstrating the app’s ease of use. Features such as the ability to communicate with others remotely (28.6%, e.g., P2-Navigator) and control the viewer’s experience via a tablet (21.4%, e.g., P3-Navigator) were particularly appreciated. Some participants (e.g., P3-Navigator, P4-Navigator) enjoyed curating their video experience, with several recommending improvements to playlist visibility, such as clearer thumbnails and labeling (see Supplementary Appendix A6 for greater detail on the feedback provided on navigational elements such as the playlist, buttons, and iconography used).

### Quality

While half of the participants (50.0%, e.g., P11-Viewer, P13-Viewer) felt the experience was immersive, particularly appreciating the ability to look around and feel engaged in the environment, many participants (42.9%) noted video resolution issues, such as blurriness or unclear texts. Still, others (e.g., P7-Viewer) reported satisfaction with the picture quality. Similarly, while a few participants (14.3%) mentioned echoing audio or challenges with competing sounds, the app’s ability to balance audio for both the viewer and navigator was seen as an asset by many participants, and the desire for more granular control over audio was acknowledged as an opportunity for improvement (see Supplementary Appendix A6 for greater detail on the feedback provided for audio, video, and connectivity functionalities).

### Content

Positive experiences were shared by those who enjoyed content on diverse themes such as holidays, music, and travel, highlighting the wide appeal of the platform. Feedback focused on expanding content offerings to include interactive games, educational materials, and personalized video experiences (P6-Viewer). One participant (7.1%) suggested adding descriptions for each video to clarify selections, while another recommended this feature to encourage navigator-viewer conversation. Participants (14.3%) also expressed interest in features that would allow them to upload personal content (P12-Viewer) or select from a more tailored library, emphasizing the technology’s potential for personalization.

The immersive nature of the VR experience was highly appreciated, with many participants describing it as “completely immersive” (P11-Viewer) and particularly valuable for building connections with family members. The ability to bond over shared experiences was highlighted by 21.4% of participants, with one participant commenting that VR could be a “lifesaver” for those separated from family (P12-Navigator).

The vast majority of participants (85.7%) indicated they would use the VR app again, with participants also expressing excitement about the potential for using VR for self-care and relaxation, with several noting the value of the experience in alleviating caregiver burnout. Many participants (64.3%) expressed that they would recommend the app to others, particularly to those who are immobile (P2-Viewer) or experiencing cognitive decline (P10-Navigator), further supporting the potential for this technology to meet diverse needs.

## Discussion

While there is a growing body of literature exploring VR for older adults, few studies have engaged with participant dyads that include older adults and their caregivers interacting together in a VR intervention. To the best of the authors’ knowledge, this study is among the few that involve dyads including older adults with cognitive impairments, such as dementia. The study sample was diverse, representing older adults with frailty and cognitive impairment, alongside their caregivers. Participants faced cognitive and physical challenges related to aging, such as forgetfulness and issues with hand dexterity (e.g., shaking hands or numbness in the fingers), which often limit functional abilities and are associated with lower technology skills in this population.^33^ Despite these challenges and lower self-reported comfort with technology, the viewer-participants found the application easy to use and reported overall satisfaction.

### Usability

Participants reported a SUS score of 72.5, comparable with other applications,^34^ suggesting acceptable usability. No serious side-effects were reported, and although some participants noted slight discomfort from the headset’s weight, they deemed it tolerable in light of the benefits of immersive VR, as has been reported in our other studies.^19^ Interestingly, two instances of cybersickness occurred for navigator-participants while using the tablet. One possible explanation is that when viewers move quickly, the mirroring of their point-of-view on the tablet can become choppy, potentially causing discomfort for the navigator when following along. In other words, the locus of control over movement in the virtual environment may be a contributing factor. Providing cautionary guidance could help minimize and mitigate these effects. On the other hand, participants expressed strong satisfaction with the ability to view what the VR user experienced via the tablet, fostering a sense of connection and shared activity.

### Navigation

Several issues were identified by participants, including difficulties with the tablet’s scroll bar and the unintuitive functionality of the ‘X’ button, which was intended to close sessions but often resulted in unintended actions, such as exiting videos instead of returning to the home screen or video library. These findings align with existing literature that highlights the challenges older adults face with scroll bars due to psychomotor limitations.^35^ Additionally, studies recommend reducing the number of scroll buttons and ensuring that navigational elements adhere to mobile app heuristics to improve usability.^36–38^ Similarly, other studies have criticized the usability of navigational buttons, suggesting that simplifying the interface by reducing the number of buttons could be beneficial.^39^ However, participant feedback in this study revealed conflicting preferences, with some participants advocating for more buttons, particularly for volume control, in contrast to recommendations for reducing visual clutter.^40^ The feedback also emphasized the need for larger elements, such as fonts and thumbnails, to improve visibility and user interaction within the app. These insights align with broader usability guidelines for designing VR applications for older adults.^1^ For example, the Accessibility for Ontarians with Disabilities Act stresses the importance of allowing users to enlarge text up to 200% while maintaining the functionality of the interface.^41^

### Quality

Participants reported that both the video and audio quality were somewhat lacking, citing issues such as blurriness, unclear text, and echoing audio in the videos, along with suggestions to improve content quality. While poor video quality was a common complaint, it is typical for 360° videos rendered into VR headsets.^42^ Achieving high resolution for an immersive experience—such as a minimum of 60 pixels per degree at a 120-Hz frame rate—requires recording 360° videos in 8K resolution or higher. However, this approach is costly, as it involves large video files that require significant processing, storage, and streaming capabilities.^42,43^ The inter-pupillary distance (IPD) setting on VR headsets also contributes to the user’s viewing comfort and depth perception.^44^ Although users can adjust the IPD themselves, improper adjustment can lead to blurry images, visual discomfort, eye strain, disorientation, and other symptoms of cybersickness.^44–46^ While automatic IPD adjustment is available on some headsets such as Varjo,^47^ this feature is still emerging and not widely available across extended reality HMDs. Participants suggested that the next iteration of the app should include a default re-centering of 360° videos whenever the headset is repositioned, ensuring the focal point is in front of the viewer. Adjusting the head strap to an optimal fit may also help improve focus and overall visual comfort.

### Content

In terms of content, participants exhibited behaviors that strongly indicated engagement and enjoyment, such as singing or dancing along with music-related videos, smiling, laughing, maintaining attentive body language and posture, and discussing the content with their partner. Participants valued the emotional bonding opportunities with family and loved ones facilitated by discussing what they would experience in VR.^25^ This “collaborative” feature has the potential to promote long-term social interaction and well-being,^48^ and participants agreed enhanced their acceptance of the system. Several participants suggested expanding the available content, with some proposing more novel features, such as integrating live experiences that would allow users to share real-time views of their surroundings through their own cameras. Notably, caregiver-participants expressed appreciation for the application’s ability to provide them with personal time for chores or self-care while ensuring their companion’s safety (i.e., respite), which addresses a critical need to alleviate caregiver strain.^49–51^ This direct affirmation from caregivers is significant, as it highlights the application’s potential to not only support the well-being of individuals living with dementia but also address the health of caregivers, a key consideration in dementia strategies where the impact of caregiver burden and burnout is increasingly recognized.

Contrary to stereotypes of aging and resistance to new technology, older adults are increasingly open to accepting VR technology,^52,53^ including HMDs.^54^ Previous research has shown that older adults are interested in experiencing “new and enriching opportunities to increase mental and social stimulation”^31^ (p. 32) through VR interventions. This study expands on these findings and contributes to the growing body of literature examining the use of VR headsets paired with tablets.^55^ Additionally, there is a well-established body of research supporting the therapeutic, recreational, and social use of VR for older adults.^56,57^ For instance, Baker et al. identified several key themes through focus groups discussing social VR interventions for older adults, including the potential for VR to facilitate novel methods of reminiscence.^58^ These discussions also highlighted the need to adapt VR applications to the aging body, taking into account age-related challenges such as reduced manual dexterity, vision changes, and declines in reasoning and processing abilities.^56,59,60^ Our study builds on these insights, particularly in its findings from the usability sessions, which emphasize the acceptability of dyadic interaction, the integration of a wide variety of immersive 360° and 2D content, and a focus on maintaining participant safety (e.g., managing cybersickness) and accessible navigation. However, this study is one of the few focusing on a dyadic VR intervention aimed at reminiscence, recreation, and socialization, contributing to the limited literature exploring VR applications for older adults when used jointly with their caregivers.

### Limitations

As this study aimed to assess the initial usability of a VR app, a small number of participants were recruited for user testing. Nevertheless, the sample is both diverse and representative of prospective end-users as it includes frail older adults with cognitive and mobility difficulties and a generally low technology literacy, along with their caregivers.

## Conclusion

This study evaluated the usability of a VR application designed for older adults and their caregivers, facilitating engagement through an HMD paired with a tablet. Following a VR2 study design, the application was developed and tested directly with target users, ensuring feedback addressed their specific needs and concerns. Unique to this study is the inclusion of participant dyads of older adults and caregivers in recreational VR usability testing, a rarity in current research. Alongside the positive reception, significant improvements are needed for navigational elements, including challenges with scrolling, volume control, and video navigation buttons. Addressing participant feedback is crucial for ensuring a safe and optimal user experience, and many of the identified challenges have known solutions that will be implemented in the next iteration of the application. Overall, our findings highlight a valuable use-case and acceptable usability for this population. The enthusiasm shown by both older adults and caregivers underscores the potential of the application for recreational and social purposes, emphasizing the benefit of facilitating shared experiences conducted even remotely.

## Notes

Supplementary Appendix A1

Supplementary Appendix A2

Supplementary Appendix A3

Supplementary Appendix A4

Supplementary Appendix A5

Supplementary Appendix A6

## Abbreviations Used

HMD: head-mounted display
IPD: inter-pupillary distance
SUS: System Usability Scale
VR: virtual reality
