# Supplementary material for: Navigating Immersion: Usability Study of a Virtual Reality Application Designed for Older Adults with Dementia and Their Caregivers
Source: J Med Ext Real. 2025 Apr 11;2(1):84–94. doi: 10.1089/jmedxr.2024.0034 (PMC13387476; doi:10.1089/jmedxr.2024.0034)
Supplement: Supplementary file 1 [file jmedxr.2024.0034_supplementary_appendix_a1.docx]

**Informed Consent Form: VR&R Usability Study** (For Participant)

Version Date: February 2024

Study Name: Evaluating the Usability of a Virtual Reality Application Designed for Older Adults and their Caregivers

Researcher: Dr. Lora Appel, 346 Stong College, 4700 Keele Street, Toronto, lappel16@yorku.ca

**Purpose of the Research:**

Virtual Reality (VR) technology displays different VR content such as films and interactive games by using images and sounds to make you feel like you are somewhere else. Research suggests that VR is enjoyable and well-tolerated among the older adult population, however, there are few VR applications that are specifically designed for older adults.

This study will test the “VR&R” (VR Rest and Relaxation) application, which has been designed for frail older adults and their caregivers. We are looking to understand how to improve the user experience in order to make future versions simpler to use and more enjoyable.

This study will use 2 technologies to view VR content: a tablet and a VR headset. See the picture below.

 **Image description**: Navigator using a tablet, and viewer seated wearing VR headset

**What You Will Be Asked to Do in the Research:**

You are being asked to take part in a research study evaluating the usability of a VR application. This study is recruiting pairs of people: One person will be an older adult (who may identify as living with frailty) who will view VR in the headset, and the other person will be a caregiver, who will control the navigation of VR from the tablet.

This session will take place either at your home or on-site at one of our community partner agencies. Participation will take approximately two hours (including taking breaks as needed) and will involve the following:

- Introduction *(15 minutes):* You will be introduced to the study, its purpose, and the research team who will walk you through the informed consent (and assent) form and answer any questions.
- Baseline Questionnaire *(15 minutes)*: You will be asked to respond to questions about yourself including demographic information (e.g., your age, gender, and experience using technology) and health history (e.g., hearing, vision, and mobility).
- Usability Testing *(60 minutes)*: As a VIEWER you will wear the VR Headset, and as a NAVIGATOR you will use the VR&R application on a tablet. A researcher will ask you, the participants, to complete various tasks/actions using the VR&R application and to **think aloud while completing them**. There will be additional researchers who will observe and take notes during the session.
- System Usability Questionnaire *(5 min):* Following the VR testing session, you will complete a brief questionnaire on paper that asks questions about the ease-of-use of the technology.
- Interview *(25 minutes)*: We will also ask you to take part in a brief interview where you can provide more in-depth feedback regarding your experience using the application, aspects you enjoyed, challenges you faced, and suggestions for improvement. At the end of the session, we will ask your permission to contact you again in a few weeks/months to complete additional test session(s) with the same format as the first session.

**Risks and Discomforts:**

Based on our prior research involving >200 older adults (including those with frailty), the risks we know of include simulator sickness (i.e., motion sickness, nausea, dizziness), which is usually mild and resolves shortly after removing the VR headset. The videos and games in the VR&R application were carefully chosen and designed to minimize the chance of simulator sickness and do not include excessive motion. Turning your head slowly while wearing the head-mounted display tends to reduce the chance of motion sickness.

Some studies have warned that unfamiliar technology, such as VR head mounted displays may be confusing to individuals with cognitive impairment and trigger disorientation or agitation. You will be asked to remove the VR headset and notify the researcher if you feel unwell at any time while using VR. Standing while using VR may cause disorientation and risk of falling. You will be asked to remain seated while using VR.

**Benefits of the Research and Benefits to You:**

There are no guaranteed benefits from participating in this study. You may find the experience of learning to use VR to be rewarding. By participating, you will help to improve the design of this VR application that is being developed as a non-drug approach to improve the wellbeing of older adults. The results of this study may lead to the development of marketable treatments or devices. Any benefit from commercial products or services will remain with the sponsor organizations.

**Voluntary Participation and Withdrawal from the Study:**

Your participation in the study is completely voluntary and you may choose to stop participating at any time, for any reason, if you so decide. For questionnaires and interviews, you may choose to skip any question you do not wish to answer by saying “pass”. Your decision to stop participating, or to refuse to answer particular questions, will not affect your relationship with the researchers, York University, or any other group associated with this project.

**Confidentiality:**

All information you supply during the research will be held in confidence and unless you specifically indicate your consent, your name will not appear in any report or publication of the research. Your contact information will be stored in a password protected file that is separate from the information you provide during this study. Personal identifiers will be removed from data files and will use participants’ alphanumeric codes instead (unique ID). Any digital data (responses to digital questionnaires) will be uploaded and stored securely on secure servers. The raw data will be accessed by a member of the immediate research team and relevant study personnel. Hard copies of data files (e.g., signed consent forms) will be safely stored in a locked facility and only research staff/research team members will have access to this information. Data files will be retained for 10 years after the end of the study and then securely destroyed. Confidentiality will be provided to the fullest extent possible by law.

The data collected in this research project may be used – in an anonymized form - by members of the research team in subsequent research investigations exploring similar lines of inquiry. Such projects will still undergo ethics review by the HPRC, our institutional REB. Any secondary use of anonymized data by the research team will be treated with the same degree of confidentiality and anonymity as in the original research project. You maintain your right to withdraw from the study at any time, including research data (if applicable).

**Costs and Reimbursement**

There are no costs or reimbursements associated with your participation in the study.

**Questions About the Research?**

If you have questions about the research in general or about your role in the study, please feel free to contact **Dr. Lora Appel either by telephone at (647) 504-6537, or by e-mail (lappel16@yorku.ca).** This research has received ethics review and approval by the Human Participants Review Sub-Committee, York University’s Ethics Review Board and conforms to the standards of the Canadian Tri-Council Research Ethics guidelines. If you have any questions about this process, or about your rights as a participant in the study, please contact the Sr. Manager & Policy Advisor for the Office of Research Ethics, 5^th^ Floor, Kaneff Tower, York University (telephone 416-736-5914 or e-mail ore@yorku.ca).

**Legal Rights and Signatures:**

I	*(participant),* consent to participate in the VR&R Usability Study conducted by Dr. Lora Appel. I have understood the nature of this project and wish to participate. I am not waiving any of my legal rights by signing this form. My signature below indicates my consent.

Signature	Date

Participant

*Signature	Date*

*(Substitute Decision Maker as necessary)*

Signature	Date

Principal Investigator/ Researcher

**Additional consent**

I consent to the use of data for future research purposes:	☐ N	☐ Y

I consent to the use of Video Recordings or Photographs:

In academic articles	☐ N	☐ Y

In print, digital and slide form	☐ N	☐ Y

In academic presentations	☐ N	☐ Y

In media	☐ N	☐ Y

In thesis materials	☐ N	☐ Y

**Assigned PARTICIPANT ID:**

**Data Collection Form - (For Participant)**

**Researcher’s Name:** ____________________ **Day:** ________________ **Time:** ___________________

**PARTICIPANT ID:**

**Role** (circle one):	Viewer (Headset User)	Navigator (Tablet User)

1. Year of Birth _______________________
2. Gender: *(Refers to how you currently identify)*

FEMALE	MALE	Prefer not to Disclose	Other: (please list)

3. Primary Language/Language of Choice: _______________________
4. Relationship to each other [e.g., parent/child, spouse, friend] _______________________
5. Dominant Hand (circle one)	LEFT	RIGHT	BOTH (ambidextrous)
6. How would you rate your vision?
   1 = Poor	2 = Fair	3 = Good	4 = Very Good	5 = Excellent
7. Do you currently wear glasses/contacts to see clearly?	YES	NO

If yes, please specify the type (e.g., bifocals/trifocals, reading, distance):

8. Do you notice any difficulty with your hearing?	YES	NO
9. Do you wear hearing aids?	YES	NO
10. Do you experience any difficulty with cognition or memory?	YES	NO

If yes, please specify any suspected/confirmed diagnosis, severity

_______________________

11. Do you experience any difficulty with using your hands or arms?	YES	NO

If yes, please describe.

_______________________

12. Do you experience any difficulty with mobility/getting around?	YES	NO

If yes, please describe (list any mobility aids used).

_______________________

13. How would you describe your comfort with technology? (Circle appropriate response.)
A) None to very limited (e.g., would have trouble turning on a device, navigating basic settings)
B) Basic/Functional (can use email, basic social media)
C) Intermediate (can use most forms of social media, strong zoom skills, strong knowledge of different online resources)
D) Advanced (prior background in IT, understand and/or can code, don't need much help with IT matters)

14. How comfortable are you with using the following technologies?

| **Device/Application** | (1)  Never used | (2)  Not at all comfortable (Used once) | (3) Somewhat comfortable (Use rarely) | (4) Reasonably comfortable (Use occasionally) | (5)  Extremely comfortable (Use frequently) |
| --- | --- | --- | --- | --- | --- |
| Simple Television (TV)  (dedicated remote: changing channels, volume) | 〇 | 〇 | 〇 | 〇 | 〇 |
| Navigating a  Smart TV | 〇 | 〇 | 〇 | 〇 | 〇 |
| Smartphone | 〇 | 〇 | 〇 | 〇 | 〇 |
| Personal desktop or laptop computer | 〇 | 〇 | 〇 | 〇 | 〇 |
| Sending Emails | 〇 | 〇 | 〇 | 〇 | 〇 |
| Social Media (facebook, twitter, instagram) | 〇 | 〇 | 〇 | 〇 | 〇 |
| Zoom/ Facetime (Video Conferencing) | 〇 | 〇 | 〇 | 〇 | 〇 |
| Online skills (e.g. shopping/ banking) | 〇 | 〇 | 〇 | 〇 | 〇 |
| Virtual Reality | 〇 | 〇 | 〇 | 〇 | 〇 |

15. Have you ever used Virtual Reality (VR) before? If YES, please describe: *(e.g., type of VR device - headset, simulation room, other; type of VR content; where, when, how, and with whom, etc.)*

16. Please add a checkmark ( ✓ ) to **the *ONE*** description that best describes how you feel.

| 〇 | You feel **very fit (for your age)**– active, energetic and motivated, exercise regularly. |
| --- | --- |
| 〇 | You **have no active disease symptoms but are less than average for your age**. you are active occasionally, e.g. seasonally. |
| 〇 | You are “managing well” - **You might have medical problems but they are well controlled,** but are not regularly active beyond routine walking. |
| 〇 | You are **not dependent on others for daily help, but often symptoms limit activities**. A common complaint is being “slowed up”, and/or being tired during the day. |
| 〇 | You have **more evident slowing, and need help in finances, transportation,** walking outside alone, medications, meal preparation, and housework. |
| 〇 | You **often have problems with stairs and need help with bathing** and might need minimal assistance (cuing, standby) with dressing. |
| 〇 | You are **completely dependent for personal car**e, from whatever cause (physical or cognitive). |

**SYSTEM USABILITY SCALE** (For NAVIGATOR ONLY - fill in after think-aloud session)

The “VR system” includes the technology you needed to use while in the VR study:

The **VR Headset** & **Tablet** and **VR&R Application**

|  | Strongly Disagree  (1) | Disagree  (2) | Neither Agree nor Disagree  (3) | Agree  (4) | Strongly Agree  (5) |
| --- | --- | --- | --- | --- | --- |
| I think that I would like to use the VR system frequently. | 〇 | 〇 | 〇 | 〇 | 〇 |
| I found the VR system unnecessarily complex. | 〇 | 〇 | 〇 | 〇 | 〇 |
| I thought the VR system was easy to use. | 〇 | 〇 | 〇 | 〇 | 〇 |
| I think that I would need the support of a technical person to be able to use the VR system. | 〇 | 〇 | 〇 | 〇 | 〇 |
| I found the various functions in the VR system were well integrated. | 〇 | 〇 | 〇 | 〇 | 〇 |
| I thought there was too much inconsistency in the VR system. | 〇 | 〇 | 〇 | 〇 | 〇 |
| I would imagine that most people would learn to use the VR system very quickly. | 〇 | 〇 | 〇 | 〇 | 〇 |
| I found the VR system very cumbersome to use. | 〇 | 〇 | 〇 | 〇 | 〇 |
| I felt very confident using the VR system. | 〇 | 〇 | 〇 | 〇 | 〇 |
| I needed to learn a lot of things before I could get going with the VR system. | 〇 | 〇 | 〇 | 〇 | 〇 |

**Observational Guide/ Notes** (For Researcher)

**Researcher’s Name:** _______________________ **Day:** ______________ **Time:** ___________________

**PARTICIPANT/s ID/s:**

**Viewer (Headset User)	Navigator (Tablet User)**

Glasses (circle)	*(Yes	No	n/a)	(Yes	No	n/a)*

Hearing Aid (circle)	*(Yes	No	n/a)	(Yes	No	n/a)*

| **Think aloud Observations:** Write out step-by-step what you see the Navigator (Tablet) and Viewer (HMD) user doing   - What experiences were selected - why? - What are the participants sharing with you? - Where do they face challenges? - What do they seem surprised by? (pleasant and otherwise) - Were explanations and hints required? - Were there tech issues (hardware/ software) that impacted session? | **Observational Notes:**  (consider the points below)**:**  1. ***Engagement*** with the Virtual Reality activity. For example:   - Shows interest and/or enthusiasm for the activity - Body language and facial expressions indicating that they are aware of the activity and/or it has caught their attention   2. ***Enjoyment*** during the Virtual Reality activity. For example:   - Smiling, laughing, and/or uplifted mood - Joking and/or playfulness   3. ***Relaxation*** during the Virtual Reality activity. For example:   - Body language and facial expressions change (e.g., grip loosening, ease of movement, settling into a comfortable position) - Sighing and/or slow breathing   4. ***Reminiscence*** during the Virtual Reality activity. For example:   - Talking about past life experiences - Talking about events/items/places that are personally meaningful   1. ***Apprehension/Anxiety*** during the Virtual Reality activity. For example:   - Body language and facial expressions indicating nervousness (e.g., anxious, fidgeting, recoiling from their surroundings) - Hesitations about their surroundings, expressed concerns for their safety   2. ***Aggression*** during the Virtual Reality activity. For example:   - Yelling, angry, or irritated tone of voice or body language - Throwing the VR headset, physically aggressive towards observer(s)   3. ***Sadness*** during the Virtual Reality activity. For example:   - Frowning and/or crying - Expressing sadness, longing, or regret |
| --- | --- |
|  |  |

# Semi-Structured Interview Script (For Researcher)

Note: All questions will be directed towards both the viewer and observer, unless otherwise specified.

*Now that you have completed the think-aloud VR session, we are hoping to learn a bit more about your experience with the technology by asking some questions.*

*To start, we want to hear about your experience in general, both the positive and the negative.*

1. How would you rate your experience using the VR&R app today? On a scale of 1-5 (1 = poor, 5 = excellent)
   a. Can you tell me a bit about anything **you liked**?
      i. What was your favorite thing (content might come up?)
   b. What did you **dislike**?
      i. Do you have any suggestions for us to improve your experience?
2. Did the experience **make you feel unwell** (e.g. headaches, dizziness, blurred vision, nausea)? On a scale of 1-5 (1 = very unwell, 5 = nothing noticed (totally well)
3. On a scale of 1-5 (1 = completely blurry, 5 =very clear) **how clear were the pictures**?
4. On a scale of 1-5 (1 = completely unclear, 5 =very clear) was the **sound in the videos clear**?
5. On a scale of 1-5 (1 = completely unclear, 5 =very clear) was **your partner’s voice clear**?
6. For VIEWER: On a scale of 1-5 (1 = very uncomfortable, 5 = very comfortable) How would you **rate the headset in terms of comfort**?
   a. Was the VR uncomfortable in any way? Prompt if: on face, pressure, weight, if holding it up with arms etc…
   b. Is there anyway you think we could improve
7. For NAVIGATOR: On a scale of 1-5 (1 = very difficult, 5 = very intuitive) How **intuitive is the application**?
   a. What was the most challenging aspect?
   b. Can you suggest any changes to the VR&R application that would make it easier to learn?
8. If you would add anything right now, to the app -what would it be?

Prompt: was anything missing?

9. Would you see yourself using the app in the future? Why/ Why not?
10. On a scale of 1 to 5, (1=not likely at all, and 5 = extremely likely), how likely is it that you would **recommend this to a friend or family member**?

Why/ Why not?

- Prompt: the TYPE of person you would recommend it to and why?

11. How would you describe VR to your friend/what would you tell them about it?
12. Is there anything else that we did not talk about today that you feel is important to add?
13. Can we contact you in a few weeks/months to participate in another test session?
