# Supplementary material for: Navigating Immersion: Usability Study of a Virtual Reality Application Designed for Older Adults with Dementia and Their Caregivers
Source: J Med Ext Real. 2025 Apr 11;2(1):84–94. doi: 10.1089/jmedxr.2024.0034 (PMC13387476; doi:10.1089/jmedxr.2024.0034)
Supplement: Supplementary file 2 [file jmedxr.2024.0034_supplementary_appendix_a3.docx]

**Participant Demographics**

| **Participant** | **Primary role** | **Age Group** | **Gender** | **Primary language** | **Dominant Hand** | **Self-reported vision** | **Difficulty in hearing** | **Clinical Frailty** |
| --- | --- | --- | --- | --- | --- | --- | --- | --- |
| 1 | Viewer | 40-44 | Female | English | Right | Not answered | No | Very Fit |
| 2 | Viewer | 70-74 | Female | English | Right | Good | Yes | Very Fit |
| 3 | Viewer | 30-34 | Female | English | Right | Very Good | No | Very Fit |
| 4 | Viewer | 90-94 | Male | English | Right | Good | Yes | Mild Frailty |
| 5 | Viewer | 95-99 | Female | English | Right | Poor | Yes | Severe Frailty |
| 6 | Viewer | 95-99 | Male | English | Right | Good | Yes | Managing Well |
| 7 | Viewer | 75-79 | Male | English | Right | Poor | Yes | Moderate Frailty |
| 8 | Navigator | 45-49 | Male | English | Right | Very Good | No | Very Fit |
| 9 | Navigator | 35-39 | Male | English | Right | Excellent | No | Very Fit |
| 10 | Navigator | 30-34 | Male | English | Right | Very Good | No | Fit |
| 11 | Navigator | 55-59 | Female | English | Right | Good | No | Very Fit |
| 12 | Navigator | 60-64 | Female | English | Right | Good | No | Severe Frailty |
| 13 | Navigator | 65-69 | Male | English | Right | Fair | No | Managing Well |
| 14 | Navigator | 75-79 | Female | English | Both (ambidextrous) | Very Good | No | Very Fit |
