# Supplementary material for: Navigating Immersion: Usability Study of a Virtual Reality Application Designed for Older Adults with Dementia and Their Caregivers
Source: J Med Ext Real. 2025 Apr 11;2(1):84–94. doi: 10.1089/jmedxr.2024.0034 (PMC13387476; doi:10.1089/jmedxr.2024.0034)
Supplement: Supplementary file 3 [file jmedxr.2024.0034_supplementary_appendix_a5.docx]

**Participant Ratings From Semi-Structured Interview**

Questions are rated from a scale of 1 (dissatisfaction) to 5 (satisfaction).

*Regarding potential side-effects, a score of 1 represents feeling unwell and 5 represents feeling well.

| **Semi-structured interview questions** | **Average rating** **(number of responses)** |
| --- | --- |
| How would you rate your experience using the VR&R app? | 4.13 (12) |
| Did the experience make you feel unwell? | 4.40* (13) |
| How clear were the pictures? | 4.20 (10) |
| Was the sound in the videos clear? | 5.00 (11) |
| Was your partner's voice clear? | 3.71 (7) |
| How would you rate the headset in terms of comfort? | 3.94 (8) |
| How intuitive is the application? | 4.21 (7) |
| How likely is that you would recommend this to a friend or family member? | 4.69 (8) |
