# Supplementary material for: Navigating Immersion: Usability Study of a Virtual Reality Application Designed for Older Adults with Dementia and Their Caregivers
Source: J Med Ext Real. 2025 Apr 11;2(1):84–94. doi: 10.1089/jmedxr.2024.0034 (PMC13387476; doi:10.1089/jmedxr.2024.0034)
Supplement: Supplementary file 4 [file jmedxr.2024.0034_supplementary_appendix_a6.docx]

#### Navigation

#### Playlist

The ability to curate a user’s experience through playlists (i.e., selecting videos in a queue) was positively received, but included recommendations for having the selected videos more apparently highlighted or having an enlarged thumbnail when selected, and tagged so their playback sequence is clear (e.g., 1st, 2nd, 3rd video). One participant (7.1%) also recommended keeping the video playlist on-screen, rather than it disappearing when adding/removing videos or editing their order. Participants enjoyed the ability to choose videos according to themes, however, two participants (14.3%) expressed that they chose videos from the “All” page of content, as it was unclear that they could select videos by theme. Three participants (21.4%) also expressed that font sizes or video icons were too small.

#### Buttons/Iconography

One participant (7.1%) was unsure how to navigate through the viewer-navigator communication functionality, including which buttons to press. Five participants (35.7%) found that the ‘X’ button did not match its assigned function in the app, as pressing it while a video was playing unintentionally closed the session instead of exiting the video as expected. Two participants (14.3%) also expressed that the ‘Back’ button was not placed or labeled intuitively. Two participants (14.3%) recommended changing the language to describe what will be closed when selecting the ‘X’ button or moving the ‘Back’ button to the left and placing the ‘X’ button to the right. Another recommendation from a participant (7.1%) in response to this was to remove the ‘X’ button and keep the ‘Back’ button while in a session to avoid confusion when attempting to leave a video. Five participants (35.7%) also expressed confusion over which volume control button affected which user (viewer in HMD, versus navigator on tablet) and whether the mute button would mute the audio in the video or mute the communication between viewer and navigator. One participant (7.1%) reported confusion around which direction the volume slider would result in an increase or decrease in volume suggesting that clarity or labels be added. Another participant (7.1%) found it unclear whether videos were 360° or 2D content.

#### Scroll

Eight participants (57.1%) reported that the scroll bar was not obvious or complained of the limited space with the bar only being located on the right side of the screen. Five participants (35.7%) suggested having the ability to use the entire screen to scroll through the app.

#### Quality

#### Video

Two participants (14.3%) reported videos starting at an off-center angle, requiring viewers to physically adjust themselves or their headsets. Almost half the participants (42.9%) presented complaints regarding poor video resolution quality, which included varied blurriness either across the screen, around the edges of the screen, unclear text/writing, or unclear resolution in nighttime scenes. Only one participant (7.1%) reported satisfaction (e.g., not bad) with the picture quality.

#### Audio

As with videos, one participant (7.1%) reported satisfaction with the audio quality, expressing that the audio level is “great when properly adjusted” (P1-Navigator). Complaints included echoing video audio when navigators and viewers were situated close to each other, as reported by two participants (14.3%), difficulties for four navigators (28.6%) to hear viewers while using their tablets, and viewers experiencing competing viewer and navigator audio as reported by six participants (42.9%).

One participant (7.1%) recommended lowering the music in videos focused on visual content, such as puppy videos, that have no relevant narration. Five participants (35.7%) suggested expanding the ability for navigators to control video, viewer, and navigator audio, such as through a “pop-up control setting” (P1-Navigator) with these volume options.

#### Connectivity

One participant (7.1%) recommended improving the headset and tablet connection, as it was found to be poor.
